# Morphological and molecular characterization of the trematodes (Digenea: Acanthocolpidae and Cryptogonimidae) of the black-spotted croaker (*Protonibea diacanthus*) (Teleostei: Sciaenidae) in northern Australia

**DOI:** 10.1017/S0031182025000502

**Published:** 2025-04

**Authors:** Megan Porter, Diane P. Barton, Xiaocheng Zhu, Shokoofeh Shamsi

**Affiliations:** 1 School of Agricultural, Environmental and Veterinary Sciences, Charles Sturt University, Wagga Wagga, NSW, Australia; 2 Gulbali Institute, Charles Sturt University, Wagga Wagga, NSW, Australia; 3 NSW Department of Primary Industries and Regional Development (DPIRD), Wagga Wagga Agricultural Institute, Wagga Wagga, NSW, Australia

**Keywords:** marine, *Orientodiploproctodaeum*, parasite, phylogeny, *Pleorchis*, sciaenidae

## Abstract

Contributing to the knowledge of digenetic trematodes in northern Australia, this study uses both morphological and molecular analysis to augment the taxonomic descriptions of existing digenean trematodes from the black-spotted croaker, *Protonibea diacanthus* (Lacepède, 1802) (Teleostei: Sciaenidae) from waters off northern Australia. Using a combination of morphological and molecular techniques, *Orientodiploproctodaeum diacanthi* Bhutta and Khan, 1970 (Digenea: Cryptogonimidae) and *Pleorchis sciaenae* Yamaguti, 1938 (Digenea: Acanthocolpidae) are compared with closely related specimens representing new geographical records of these species, and contributing the first phylogenetic analysis of both digenean species. Both *O. diacanthi* and *P. sciaenae* were genetically distinct from other reported specimens of the respective families Cryptogonimidae and Acanthocolpidae, based on phylogenetic results and the supporting morphological descriptions from past publications. Despite the conclusive findings in this study, the species presented in the phylogenetic analyses lack sequences across a range of genes, leading to difficulties in deciphering the phylogenetic and evolutionary relationships of many species and highlighting the need for future research to improve species-level identification of parasites in Australian waters.

## Graphical abstract

**Figure:**
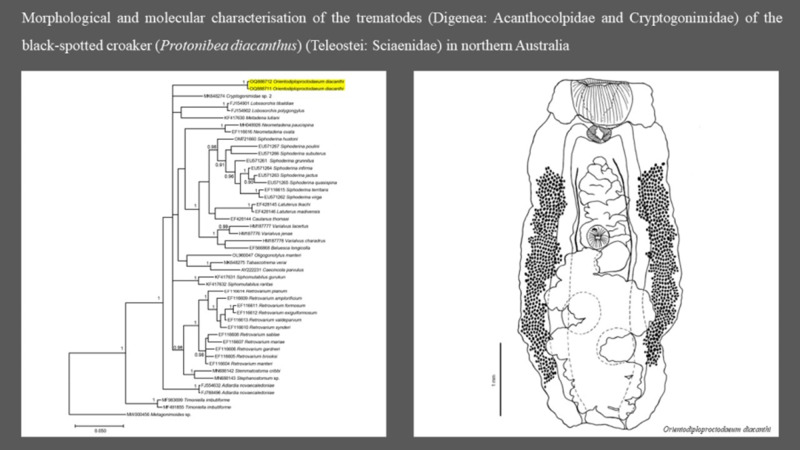

## Introduction

There remains a large gap regarding the knowledge of the overall biology and ecology of many important marine fish species of Australia (Hobday et al., 2019). Disease emergence and human-mediated climate changes, in addition to habitat decline from water pollution, and the facilitated introduction of terrestrial pathogens to aquatic ecosystems (Ward and Lafferty, 2004) are stressors in addition to impacts from over-fishing (Hobday et al., 2019). Details on the baseline levels and diversity of parasites and disease presence is required, from fish hosts across the Indo-Pacific region, to build on the biology of fish to enable sustainable management of important fisheries.

Like many large-bodied marine fish species, *Protonibea diacanthus* (Lacepède, 1802) (Teleostei: Sciaenidae) is vulnerable to population crashes (Taillebois et al., 2017). Recent developments in the fisheries management of *P. diacanthus* incorporated parasites into a determination of stock structure (Taillebois et al., 2017). This has led to a subsequent series of reports utilising the parasite assemblage to determine aspects of host and parasite biology and ecology (Porter et al., 2023b, 2023c, 2024). Detailed examination of the parasite fauna determined a number of species of parasites, which have subsequently been described from across northern Australia, including the nematodes *Philometra protonibeae* (Moravec and Barton, 2015), and *Philometroides stomachicus* Moravec and Barton, 2016; the pentastomes *Alofia merki* Giglioli in Sambon, 1922; *Sebekia purdieae* Riley et al., 1990, *Sebekia* sp. 2, and *Sebekia* sp. 3, and more recently, the monogeneans *Diplectanum timorcanthus* Porter et al., 2023 and *Diplectanum diacanthi* Porter et al., 2023 and the copepod *Lernanthropus paracruciatus* (Moravec and Barton, 2015; Barton and Morgan, 2016; Boxshall et al., 2020; Porter et al., 2023a).

Despite species descriptions of pentastomes, philometrids, monogeneans and copepods from marine fishes off northern Australia, the list of parasite species from *P. diacanthus* in Taillebois et al. (2017) and Porter et al. (2023c) did not include identified species of digeneans, although many were identified to genus. No digeneans have yet been identified at the species level from *P. diacanthus* in Australian waters (Beumer et al., 1986; Lester and Sewell, 1989). This study presents an integrated morphological and molecular description of two species of digenetic trematodes found in *P. diacanthus* in Australian waters.

## Materials and methods

### Fish and parasite collection

Specimens of *P. diacanthus* were collected from several locations across northern Australia, including in WA: Roebuck Bay, Camden Sound and Wyndham, and in NT: Wadeye, Peron Islands, Melville Island, Bathurst Island, Outer Darwin Harbour, Sampan Creek, Maningrida, Vanderlin Islands and Arafura Sea. Fish were collected using hook and line capture techniques by staff of both the Western Australian and Northern Territory Departments of Fisheries, Indigenous Marine Rangers and by commercial fishers. Fish were euthanized *via* percussive stunning (Charles Darwin University Animal Ethics Approval #A13014 and #A19009), placed on ice and transported to the laboratory for processing; all samples were frozen prior to examination for parasites. After thawing, the stomach, pyloric caecae and intestinal tract were separated from the mesenteries and associated organs and opened longitudinally along its length for examination. Digeneans found were collected and preserved in 70% ethanol until further processing.

### Morphological examination

Digenean specimens selected for morphological analysis, including the specimens also used for molecular analysis, were stained with acetocarmine, dehydrated through a graded ethanol series, cleared in xylene, and mounted in Canda balsam. Measurements of characters of systemic importance were obtained with the use of both a Nikon DS-Ri2 motorized microscope and/or a calibrated eyepiece from a compound microscope. Measurements are in micrometres as a mean value followed by a range in parentheses (if provided). The mean values are compared to previous descriptions and synonyms for *Orientodiploproctodaeum diacanthi* and *Pleorchis*, and are provided in Tables 1 and 2, respectively. The terms forebody and hindbody follow the definition of Bartoli et al. (2004). All drawings were made with the use of a Nikon Y-IDT drawing tube which was mounted on a Nikon Eclipse E200 microscope.

### Molecular identification

From the specimens chosen for molecular study, a small posterior segment of the parasites was sliced aseptically (prior to staining) and frozen for molecular processing in a 1.5 mL micro- tube. Genomic DNA was extracted from the parasite samples using the DNeasy Blood & Tissue Kit (Qiagen, Melbourne, Australia), following a modified protocol (Shamsi et al., 2018) and eluted in 40 µL of elution buffer. Polymerase chain reaction (PCR) amplification of different gene regions was performed for *O. diacanthi* and *P. sciaenae*, with protocols for molecular analysis differing for each genus. For *O. diacanthi*, the nuclear 28S rRNA gene was amplified using the primer set LSU5 (5’ – TAGGTCGACCCGCTGAAYTTAAGCA – 3’) and ECD2 (5’ – CCTTGGTCCGTGTTTCAAGACGGG – 3’) (Miller and Cribb, 2007a), the ITS1 region was amplified using the primer set BD1 (5’ – GTCGTAACAAGGTTTCCGTA – 3’) and 4S (5’ – TCTAGATGCGTTCGAARLTGTCGATG – 3’), and the ITS2 region was amplified using the primer set 3S (5’ – GGTACCGGTGGATCACGTGGCTAGTG – 3’) and ITS2.Sr (5’ – CCTGGTTAGTTTCTTTTCCTCCGC – 3’) (Miller and Cribb, 2007a, 2007c). PCR was performed in a 25 µL reaction containing 4 µL template DNA, 1X GoTaq® Flexi Buffer, 2.5 mM of MgCl_2_, 0.4 mM of each dNTP 0.2 mM of each primer and 1.25 U of GoTaq® Flexi DNA Polymerase. The amplification cycle for the 28S rRNA and ITS2 regions had an initial denaturation at 96^o^C for 5 min, followed by 25 and 40 cycles of amplification, respectively: denaturation at 96^o^C for 1 min, annealing at 54^o^C for 15 s, extension at 72^o^C for 30 s; and a final extension at 72^o^C for 4 min. The ITS1 region was amplified with an initial denaturation at 95^o^C for 5 min, followed by 40 cycles of amplification: denaturation at 95^o^C for 30 s, annealing at 55^o^C for 30 s, primer extension at 72^o^C for 1 min; and a final extension at 72^o^C for 10 min. For *P. sciaenae,* the nuclear 18S rRNA gene was amplified using the primer set 1100 F (5’ – CAGAGATTCGAAGACGATC – 3’) and wormB (5’ – CTTGTTACGACTTTTACTTCC – 3’) (Littlewood and Olson, 2001), and the nuclear 28S rRNA gene was amplified using the primer set LSU5 and ECD2 (as with *O. diacanthi*) (Olson et al., 2003). The amplification cycle for both regions had an initial denaturation at 95^o^C for 2 min, followed by 40 cycles of amplification: denaturation at 95^o^C for 30 s, annealing at 50^o^C for 30 s, primer extension at 72^o^C for 45 s; and a final extension at 72^o^C for 10 min. An aliquot (2.5 µL) of each amplicon was examined on a 1.5% w/v agarose gel, stained with GelRed^TM^ and photographed upon transillumination.

PCR amplicons of representative samples were sent to the Australian Genome Research Facility (AGRF) and were subjected to Sanger sequencing using the primer sets as for PCR.

Slide-mounted and unmounted specimens will be deposited in the collections of the Museum and Art Gallery of the Northern Territory (MAGNT), Queensland Museum (QM), the South Australian Museum (AHC: Australian Helminthological Collection) and the Western Australian Museum (WAM). Voucher numbers are available for specimens currently deposited.

### Construction of phylogenetic tree

Sequence data including chromatograms of forward and reverse AB1 trace files, were observed initially through Sequence Scanner Software 2 (Applied Biosystems® Genetic Analysers). Subsequently, sequences were aligned using BioEdit 7.2.0 (Hall, 1999) with sequences of closely related species and outgroups for each region from GenBank (Supplementary Table S1 and Supplementary Table S5). The pairwise genetic distance was calculated using MEGA version 11 (Tamura et al., 2021), indels were pairwise delated for analysis.

For sequences of the species from the family Cryptogonimidae the alignments were truncated to 729, 931 and 450 bp for 28S, ITS1 and ITS2 gene regions, respectively. For sequences of the species from the family Acanthocolpidae the alignments were truncated to 941 and 826 bp for 28S and 18S rRNA regions, respectively. Phylogenetic analyses were performed as described in Barton et al. (2022). The best fit evolutionary model as inferred by jModelTest 2 (Darriba et al., 2012). For *Orientodiploproctodaeum* sp., GTR + I + G model was chosen for the 28S rRNA region, while the GTR + G model was used for the ITS1 and ITS2 regions; and for *Pleorchis sciaenae* the GTR + I + G and K80 + I + G models were selected for 28S and 18S rRNA regions, respectively. Outgroup species were selected from species from the same superfamily but different family. *Metagonimoides* sp. (MW000456), *Dexiogonimus ciureanus* (AY245702) and Heterophyidae sp. (MZ825158) were selected as outgroup for 28S, ITS1 and ITS2 phylogenetic analyses for *Orientodiploproctodaeum* sp., respectively (Supplementary Table S1). *Brachycladium goliath* (KR703279) was used as outgroup for the phylogenetic analysis of *Pleorchis sciaenae* at both 28S and 18S rRNA gene regions to assess the relationship of *Pleorchis sciaenae* with other acanthocolpids (Supplementary Table S5). The analyses were carried out using MrBayes 3.2.7 (Ronquist and Huelsenbeck, 2003), the MCMC runs were performed for 2000 000–400 0000 generations until the standard deviation of split frequences below 0.01. The first 50% of the sampled trees were discarded as burn-in. The confidence of phylogenetic grouping was estimated using posterior probabilities. The tree was visualized using Figtree v1.4.3 (Rambaut, 2009).

## Results

### Infection dynamics

Across the studied sample of *P. diacanthus* for this manuscript, *Orientodiploproctodaeum diacanthi* had an overall prevalence of 93.42% infection, with a mean intensity of 28.47 (1‒588). *Pleorchis sciaenae* had an overall prevalence of 16.44% infection in *P. diacanthus*, with a mean intensity of 4.05 (1‒37). The parasites identified in this study are described here.

### Descriptions and remarks (including descriptive morphological features)

**Class Trematoda**

**Subclass Digenea**

**Order Plagiorchiida**

**Family Cryptogonimidae Ward, 1917**

**Genus *Orientodiploproctodaeum* Bhutta and Khan, 1970**

**Figure 1.**
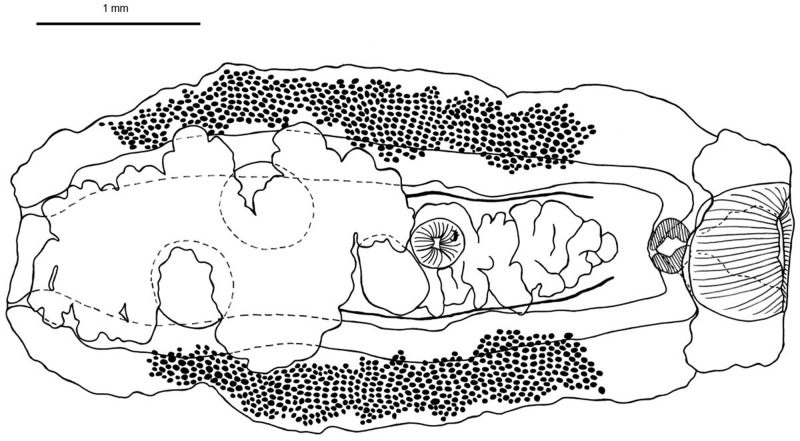
Line drawing of a mature *Orientodiploproctodaeum diacanthi* (ventral view) collected from *Protonibea diacanthus* from Northern Territory. Scale bar 1000μm. Gonotyl represented by dark mark on the anterior surface of the ventral sucker.

***Orientodiploproctodaeum diacanthi* Bhutta and Khan, 1970** (Figure 1)

*Synonyms: Anterodiscus biseminalis* Bilqees, 1974*. Anterodiscus triuteri* Bilqees, 1974. *Cryptocollaritrema provesiculatum* Madhavi, 1976*. Folliculovarium indicum* Singh and Sinha, 1981*. Harutrema marinum* Mehra and Kharoo, 1975*. Multiovarium heteroformis* Bilqees, 1974*. Multiovarium interruptum* Bilqees, 1974*. Orientodiploproctodaeum heteroformis* (Bilqees, 1974). *Orientodiploproctodaeum indicum* (Singh and Sinha, 1981)*. Orientodiploproctodaeum interruptum* (Bilqees, 1974)*. Orientodiploproctodaeum provesiculatum* (Madhavi, 1974). *Orientodiploproctodaeum triuteri* (Bilqees, 1974).

*Host: Protonibea diacanthus* (Lacepède, 1802) (Teleostei: Sciaenidae) ‘Black Jewfish’ or ‘Black-spotted croaker’.

*Locality*: Timor Sea (including Beagle Gulf and Van Diemen Gulf), Northern Territory, Australia

*Site in host*: Pyloric caeca

*GenBank accession*: 28S (OQ888711-OQ888712), ITS1 (OQ888688-OQ888689), ITS2 (OQ888737-OQ888739).

*Redescription* (Figure 1):

Body robust, broadly ovate, longer than wide. Forebody occupies 37.33% of body length. Tegument thick; spines not observed. Large anterior muscular collar present in oral sucker region. Oral sucker large; transversely oriented oblate ovoid, flattened dorsoventrally. Pre-pharynx short. Pharynx large, longitudinally ovate. Oesophagus short. Intestinal bifurcation immediately post-pharynx. Intestinal caeca pass wide, open out through separate anal pores. Ventral sucker small, subglobular, on ventral surface. Testes two large ovate, oblique, in anterior part of hind body, outlines often obscured by vitelline. Seminal vesicle long, coiled; extending from intestinal bifurcation in forebody to ventral sucker. Gonotyl immediately anterior to ventral sucker. Ovary not distinctly observed, at midbody immediately anterior and adjacent to testes. Seminal receptacle large ovate, between seminal vesicle and ovary when observed. Ovary pretesticular, large when observed. Vitelline follicles small, in two wide lateral zones of body, extend from level of seminal vesicle to below level of posterior testis. Uterus coils extensively from level of ventral sucker throughout entire hind body. Eggs small, darkly tanned.

### Morphological remarks

The specimens collected in this study were identified as *O. diacanthi* based on the overall morphological similarity with the previous description by Bray (1987): the oral sucker surrounded by a large muscular anterior collar, body not exceptionally elongate, two testes, gonotyl positioned anteriorly to the ventral sucker, and the caeca opening via separate ani at the posterior extremity. The original description by Bhutta and Khan was based upon three specimens and Bray (1987) examined a further three unflattened specimens, in addition to slide mounted material originally described under different genera, in his redescription.

Specimens described here are differentiated from the closely related species *O. chinabutae* by the presence of a genital pore and its associated sac. Additionally, *O. diacanthi* lacks the prostatic gland cells that are described as present in *O. chinabutae.* Previous comparisons by Bray (1987) also described *O. chinabutae* to be of much larger size than *O. diacanthi* which, although questionable based on the small number of specimens for the description of *O. chinabutae* (two), the present study also found much smaller specimens in total body length (2950 μm; *O. chinabutae* 7200 μm) (Table 1). *Orientodiploproctodaeum chinabutae* can also be differentiated from *O. diacanthi* and synonyms based on the oral sucker to ventral sucker ratios, with the oral sucker being approximately double the size of the ventral sucker in all descriptions except for *O. chinabutae* which reports the oral sucker to be four times larger than the ventral sucker (Table 1). Further to this, the overall oral sucker size is much greater in *O. chinabutae* (920 μm long, 1050 μm wide) when compared to *O. diacanthi* in this study (470 μm long, 620 μm wide).

**Table 1.** Comparative measurements of *Orientodiploproctodaeum diacanthi* and synonyms (identified with asterisks). measurements all in micrometres, expressed as a mean; a dash (‒) indicates that measurements could not be made or were not available

Species	*Orientodiploproctodaeum diacanthi* (Bhutta and Khan, 1970)	*Orientodiploproctodaeum diacanthi* (Bhutta and Khan, 1970)	Multiovarium heteroforme*	Multiovarium interruptum*	Cryptocollaritrema provesiculatum*	*Orientodiploproctodaeum chinabutae* (Bray, 1987)
Reference	Present study	Bray, 1987	Bray, 1987	Bray, 1987	Bray, 1987	Bray, 1987
Host	*Protonibea diacanthus* (Sciaenidae)	*Protonibea diacanthus* (Sciaenidae)	*Protonibea diacanthus* (Sciaenidae)	*Protonibea diacanthus* (Sciaenidae)	*Lutjanus* sp. (Lutjanidae)	*Nibea soldado* (Sciaenidae)
Location	Australia: Northern Territory	Pakistan: Karachi Coast	Pakistan: Karachi Coast	Pakistan: Karachi Coast	Bay of Bengal	Thailand: Chao Phraya River
No. measured	3 22	3	1	1	1	1
Total length	2950 (1710–5030)	2210–2670	4100	3100	5200	7200
Width	1350 (930–2220)	1340–1420	1700	1570	1780	2680
Forebody as % of body-length	37.33	31.75	33.70	31.00	33.10	40.60
Anterior collar length	490 (220–860)	450–570	860	490	1220	1100
Anterior collar width	1170 (840–1620)	640–960	960	380	1670	1670
Oral sucker length	470 (270–710)	280–390	520	330	540	920
Oral sucker width	620 (420–880)	470–540	550	540	570	1050
Ventral sucker length	230 (100–330)	170–190	230	180	220	250
Ventral sucker width	250 (90–360)	190–230	260	200	250	280
Sucker ratio length	1: 0.49	1: 0.53	1: 0.44	1: 0.55	1: 0.41	1: 0.27
Sucker ratio width	1: 0.40	1: 0.41	1: 0.47	1: 0.37	1: 0.44	1: 0.27
Pharynx length	190 (100–300)	190–220	230	190	180	250
Pharynx width	240 (100–490)	180–190	280	230	270	700
Anterior Testes length	290 (150–480)	‒	430	290	430	‒
Anterior Testes width	350 (180–800)	250	240	230	430	260
Posterior Testes length	310 (150–530)	‒	380	300	490	‒
Posterior Testes width	320 (100–640)	250	280	230	440	280
Ovary length	310 (250–400)	‒	310	200	590	‒
Ovary width	520 (450–600)	‒	650	540	1160	‒
Eggs length	11 (7–12)	14–16	14	16	14	17
Eggs width	10 (7–12)	10–12	12	11	12	9

### Molecular remarks

**Figure 2.**
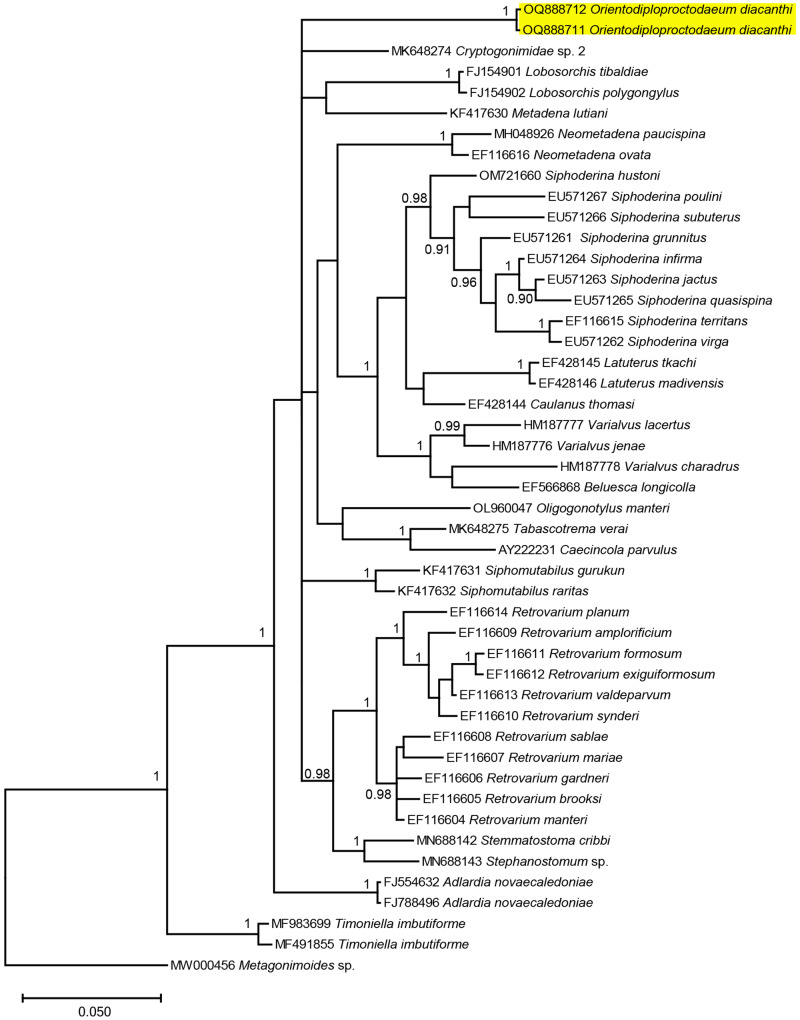
Phylogenetic tree based on the Bayesian phylogenetic relationships of *Orientodiploproctodaeum diacanthi* (this study, highlighted in yellow) and other species of the family Cryptogonimidae inferred from 28S rRNA (partial) sequences, available from GenBank (Supplementary Table S1). Clade posterior probability (> 0.90) is indicated at nodes.

**Figure 3.**
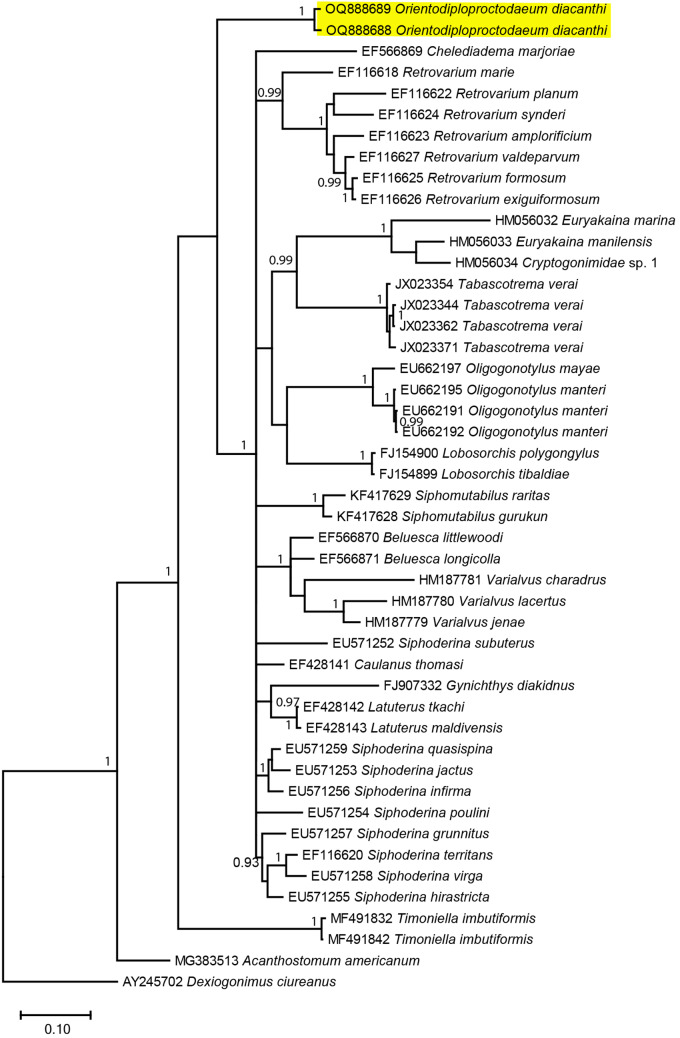
Phylogenetic tree based on the Bayesian phylogenetic relationships of *Orientodiploproctodaeum diacanthi* (this study, highlighted in yellow) and other species of the family Cryptogonimidae inferred from ITS1 sequences, available from GenBank (Supplementary Table S1). Clade posterior probability (> 0.90) is indicated at nodes.

**Figure 4.**
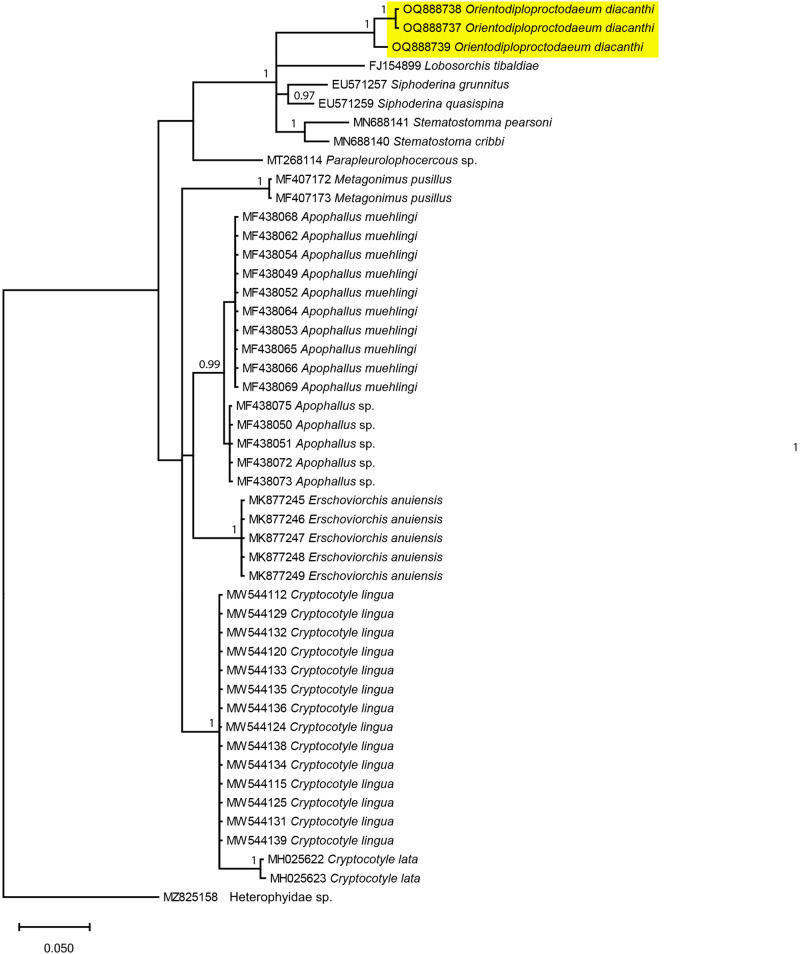
Phylogenetic tree based on the Bayesian phylogenetic relationships of *Orientodiploproctodaeum diacanthi* (this study, highlighted in yellow) and other species of the family Cryptogonimidae inferred from ITS2 sequences, available from GenBank (Supplementary Table S1). Clade posterior probability (> 0.90) is indicated at nodes.

The results for the molecular sequences showed that our *O. diacanthi* specimen was distinct from other reported parasites of the Cryptogonimidae family (Figures 2–4), with minimum among species differences being 6.65%, 11.56% and 8.62% at 28S rRNA, ITS1 and ITS2 regions, respectively (Supplementary Table S2, Supplementary Table S3 and Supplementary Table S4). Our *O. diacanthi* specimens formed a cluster distinct from other Cryptogonimidae species at the 28S and ITS1 regions. In the analysis of the ITS1 region, more than half of the published sequences making up the analysis are from marine fish hosts collected in northern Queensland (Figure 3, Supplementary Table S1). Our *O. diacanthi* specimens remained in a separate cluster to these Australian records, however grouped with Australian records of Cryptogonimidae at the ITS2 region, appearing separated from the sequences of Cryptogonimidae collected from a variety of hosts from European locations (Figures 4, Supplementary Table S1). Although grouped more closely to certain specimens from Australian hosts (Figures 3 and 4), results support the morphological findings with *O. diacanthi* consistently dissimilar to those specimens with previously published sequences. For the specimens within the clade, *O. diacanthi* is morphologically distinct from the other genera with *Siphoderina* Manter, 1934*, Stemmatostoma*, and *Lobosorchis* Miller & Cribb, 2005 all possessing large oral spines, and a number of other distinguishing characteristics (Miller et al., 2009a; Miller and Adlard, 2020; Martin and Cutmore, 2022).

**Class Trematoda**

**Subclass Digenea**

**Order Plagiorchiida**

**Family Acanthocolpidae, Lühe, 1909**

**Genus *Pleorchis*, Railliet, 1896**

**Figure 5.**
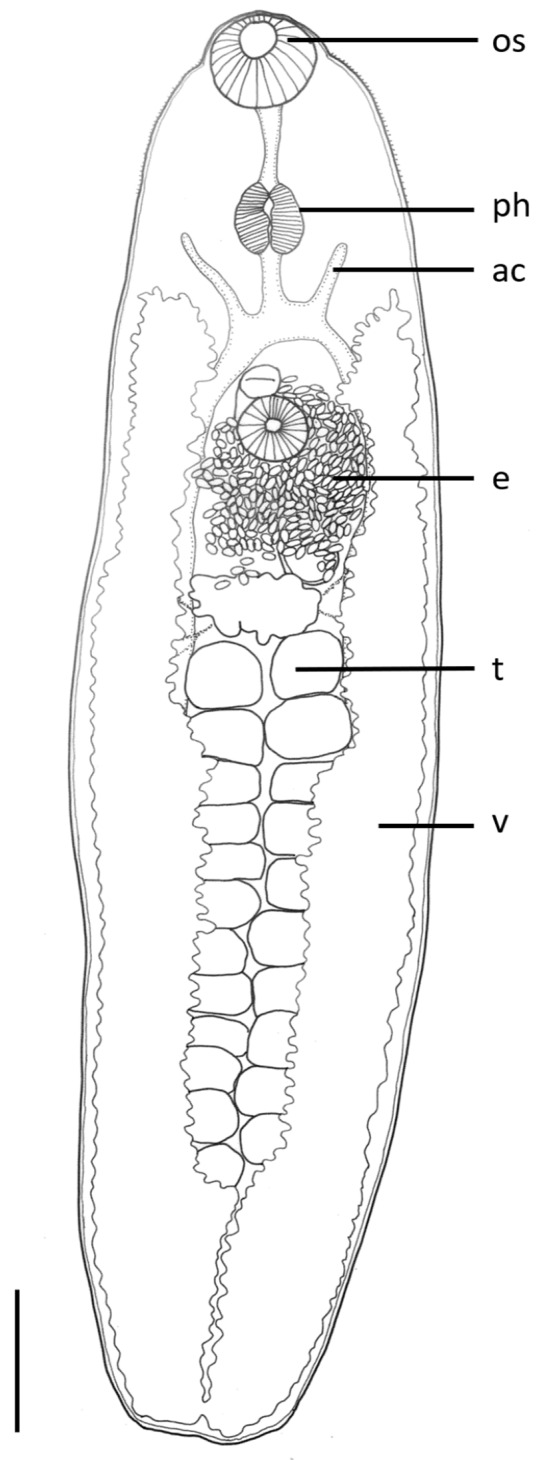
Line drawing of a mature *Pleorchis sciaenae* (ventral view) collected from *Protonibea diacanthus* from melville island, northern territory. Scale bar 500 μm. AC, Anterior Caecum; E, Eggs in uterus; OS, Oral Sucker; PH, Pharynx; T, Testis; V, Vitellaria Follicles.

**Figure 6.**
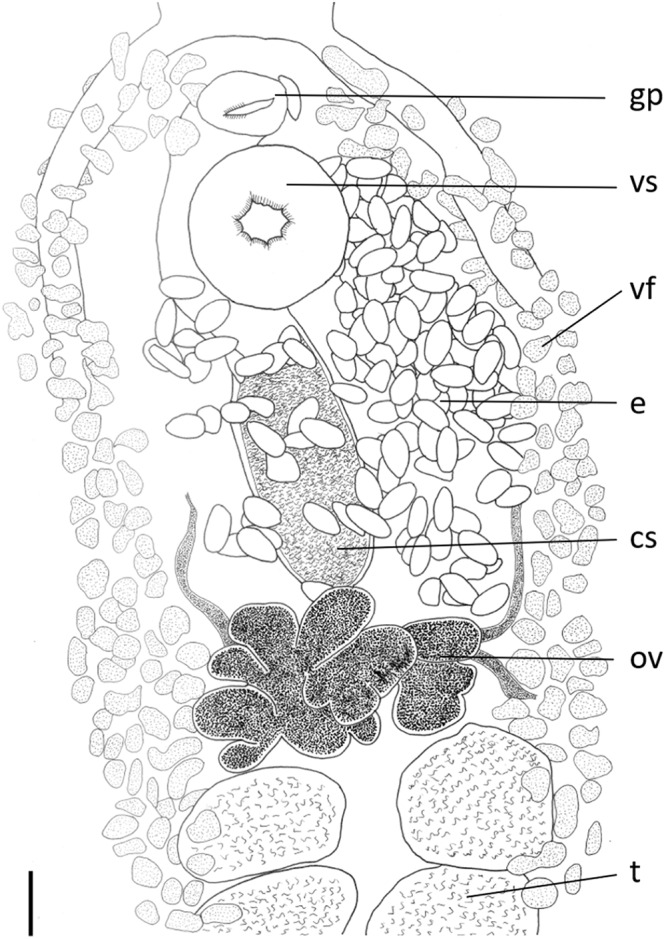
Line drawing of the reproductive system of a specimen of *Pleorchis sciaenae* (ventral view). scale bar 100μm. CS, Cirrus Sac; E, Egg; GP, Genital Pore; OV, Ovary; T, Testis; VF, Vitelline Follicle; VS, Ventral Sucker.

*Pleorchis sciaenae***, Yamaguti, 1938 (Figure 5 and Figure 6)**

*Synonyms: Parapleorchis keshavai* (Gupta et al., 2011); *Parapleorchis puriensis* (Gupta and Ahmad, 1976) Al-Yamani and Nahhas, 1981; *Pleorchis ghanensis* (Fischthal and Thomas, 1968): *Pleorchis psettodesai* Gupta and Gupta, 1978; *Pleorchis puriensis* Gupta and Ahmad, 1976.

*Host: Protonibea diacanthus* (Lacepède, 1802) (Teleostei: Sciaenidae) ‘Black Jewfish’ or ‘Black-spotted croaker’.

*Locality*: Roebuck Bay, Camden Sound, Wyndham (Western Australia); Wadeye, Bynoe Harbour, Outer Darwin Harbour, Cape Hotham, Bathurst Island, Melville Island, Maningrida, Arafura Sea, Gove, Groote Eylandt, Vanderlin Islands, Northern Territory, Australia.

*Site in host*: Pyloric caeca, intestine

*Voucher specimens deposited*: MAGNT (D001881‒001889), AHC (36880‒36884), WAM (V11015‒11018), QM (G239131‒G239134).

*GenBank accession*: 18S (MZ662944), 28S (MZ662945).

*Description* (Figures 2 and 3; measurements based on 31 adult specimens):

Body flat, elongate with maximum width at level of anterior testes, rounded anteriorly, slightly truncate posteriorly, with mid-terminal notch. Pre-oral lobe indistinct. Tegument. Body surface spined. In specimens where spines are obvious, spines large and dense in anterior third of body, becoming progressively thinner and less dense towards posterior extremity. Spines present as far as mid testes zone. Most specimens with few spines present/obvious; staining of some specimens show ‘notches’ where spines were present but appear to have fallen out. Suckers. Oral sucker slightly ventro-subterminal, round with small aperture. Ventral sucker round, at approximately one-quarter of body length from anterior end. Oral sucker larger than ventral sucker by ratio 1.4 (1.2–1.6). Prepharynx present. Pharynx approximately spherical, slightly larger than ventral sucker; anterior third of different appearance, markedly narrower in dimension to rest of pharynx (anterior circular muscular ring of Bray, 1986). Oesophagus short. Intestine H-shaped; intestinal bifurcations in forebody; anterior caeca simple, thinner than posterior caeca, reaching to anterior edge of pharynx; posterior caeca simple, reach to posterior end of body, usually difficult to discern as obscured by vitelline follicles in mature specimens. Testes numerous, intercaecal, aligned in 4 parallel rows, 2 ventral and 2 dorsal; dextral rows separated from sinistral rows by excretory vesicle; number of testes not always clear as they can be obscured by vitelline follicles; mean number of testes 43 (range 40–46), with following variations: ventral testes: right 10–12, left 9–12; dorsal testes: right 8–13, left 9–12. Testes subglobular, occasionally indented, with anterior testes slightly larger than posterior, extend from immediately posterior to ovary to 678.6 (440–1020) from posterior end [approximately 15% (12–20%) of body length from posterior end]. Testicular rows occupy approximately 42% (35–47%) of body length. Cirrus sac always overlaps dextral side of ventral sucker, extends into hindbody to level midway between ventral sucker and ovary, curved, broader proximally, contains bipartite seminal vesicle. Internal structure often obscured by uterus. Ejaculatory duct rectilinear, opens into genital atrium; when protruded, short, unarmed. Genital atrium spherical, wide, thin-walled. Genital pore large, median, at level of intestinal bifurcation, slightly anterior to anterior margin of ventral sucker. Ovary in form of 7 (5–8) more or less developed lobes, wider than long, slightly anterior to mid-body, generally completely anterior to testes. Uterus pre-ovarian, inter-caecal (slightly overlaps caeca in individuals with many eggs), coils and fills space between ovary and ventral sucker, ventral to cirrus sac. Eggs thin-shelled, with a small protrusion evident at one end in many eggs, often collapsed in mounted specimens, yellowish. Vitellarium follicular; follicles small, very numerous, extend from level slightly behind posterior edge of pharynx (approximately 18% (14–23%) of body length from anterior end) to posterior extremity of body and to lateral body margins, sometimes merging anterior to ventral sucker, totally confluent in dorsal and ventral post-testicular space. Anterior and posterior longitudinal vitelline ducts on each side of body unite to form transverse vitelline duct dorsal to ovary. Excretory vesicle tubular, extends forward as far as ovary, located between 4 rows of testes. Excretory pore terminal, within terminal notch of body.

### Morphological remarks

The specimens collected in this study were identified as *P. sciaenae* based on the overall morphological similarity with previous descriptions of *P. sciaenae* (Table 2). Overall, the measurements of *P. sciaenae* collected in this study were closest to the measurements for *P. sciaenae* reported by Nahhas et al. (1998) collected from *Otolithes argenteus* from the Kuwaiti coast, Arabian Gulf. The overall body size and ratios of various measurements (for example, forebody to total length, oral sucker length to ventral sucker length of testes zone) were very similar to the previous descriptions of *P. sciaenae*. However, the measurements of the testes dimensions could not be compared to *P. sciaenae*.

**Table 2.** Comparative measurements of *Pleorchis sciaenae* in comparison to other members of ‘group 2’ of Bartoli et al. (2004), with the exception of *P. puriensis* for which no data were available. Measurements all in micrometres, expressed as a mean and with range in parentheses if applicable; percentages (%) calculated as percentage of total length of individual; a dash (‒) indicates that measurements could not be made or were not available

Species	*Pleorchis sciaenae*	*Pleorchis sciaenae*	*Pleorchis sciaenae*	*Pleorchis ghanensis*	*Pleorchis hainanensis*	*Pleorchis polyorchis*
Reference	Present study	Bartoli et al., 2004^a^	Nahhas et al., 1998	Fischthal and Thomas, 1968	Bartoli et al., 2004	Bartoli et al., 2004
Host	*Protonibea diacanthus* (Sciaenidae)	*Nibea albiflora* (Sciaenidae)	*Otolithes argenteus* (Sciaenidae), accepted as *Otolithes ruber* (Bloch & Schneider, 1801)	*Cynoscion macrognathus; Pomadasys jubelini* (Sciaenidae)	*Pennahia anea* (Lutjanidae)	*Sciaena umbra* (Sciaenidae)
Location	Australia: Northern Territory	East China Sea	Arabian Gulf: Kuwaiti coast	Tema, Ghana	China	Corsica: France
No. measured	31	4	5	8	1	10
Total length	4417 (2840–6060)	2700–3100	5430 (3425–6500)	4660–5247	6379	2971 (2244–4076)
Width	1076 (740–1530)	750–850	1696 (1250–1930)	1310–1550	1069	911 (680–1138)
Forebody	1095 (740–4530)	588	‒	878–1235	1650	828 (533–1074)
Forebody as % of body-length	25 (21–29)	21^b^	33 (30–36)	19–24^b^	26^b^	28^b^
Hindbody	3118 (1910–4380)	2200	‒	3145–3995	4690	1917 (1438–2749)
Hindbody as % of body-length	70 (65–6)	80^b^	66 (58–68)	67–76^b^	74^b^	64 (60–68)
Oral sucker length	303 (204–400)	170–188	314 (180–392)	258–310	351	294 (316–412)
Oral sucker width	338 (253–420)	210–250	362 (268–420)	276–354	‒	335 (276–412)
Ventral sucker length	218 (163–280)	185–210	251 (205–300)	224–298	217	231 (184–317)
Ventral sucker width	229 (163–300)	‒	265 (220–300)	218–310	‒	249 (203–343)
Oral sucker length: Ventral sucker length	0.73 (0.63–0.84)	0.9–1.1	0.76 (0.66–0.86)	0.83–1.02	0.62	0.79 (0.70–0.97)
Prepharynx length	203 (100–320)	75–130	230 (160–310)	155–346	367	123 (13–203)
Pharynx length	232 (163–300)	150–175	238 (170–275)	165–250	184	222 (184–292)
Pharynx width	222 (155–290)	150–160	275 (200–320)	195–236	200	236 (210–349)
Pharynx to Ventral sucker	357 (122–560)	‒	‒	‒	‒	182 (64–254)
Anterior end to anterior limit of vitellarium	807 (505–1200)	‒	‒	‒	‒	539 (317–667)
Cirrus Sac length	733 (114–978)	400–550	703 (560–900)	563–766	618	434 (330–608)
Cirrus Sac width	193 (120–277)	100–120	159 (125–200)	133–177	84	113 (83–170)
Proximal Seminal Vesicle length	382 (204–560)	‒	‒	188–386	251	94 (63–154)
Distal Seminal Vesicle length	116 (82–163)	‒	‒	122–224	‒	110 (40–144)
Ovary length	264 (163–391)	180–250	400 (345–600)	246–416	367	216 (115–330)
Ovary width	398 (228–587)	250–400	600 (400–730)	391–525	468	347 (240–543)
No. of lobes ovary	7 (5–8)	‒	‒	‒	‒	8 (6–9)
Ventral sucker to Ovary	384 (204–611)	‒	‒	331–452	‒	198 (114–273)
Ventral sucker to Anterior Testes	575 (350–852)	‒	‒	‒	‒	375 (235–560)
Anteriormost Testes length	186 (82–300)	‒	‒	132–220^c^	‒	109 (63–192)
Anteriormost Testes width	211 (98–340)	‒	‒	140–254^c^	‒	‒
Posteriormost Testes length	184 (114–300)	‒	‒	‒	‒	86 (40–154)
Posteriormost Testes width	179 (82–320)	‒	‒	‒	‒	‒
Total length of Testes rows	1849 (1000–2600)	‒	‒	‒	‒	977 (667–1422)
Testes Zone %	42 (35–47)	‒	‒	‒	‒	‒
Post-Testes length	679 (440–1020)	‒	‒	820–960	‒	573 (406–984)
Post-Testes %	15 (12–20)	‒	‒	18^b^	‒	19
No. of Testes	43 (40–46)	‒	44	44 (44–46)	44	44 (40–45)
Eggs length	63 (54–70)	69–72	70 (65–75)	61 (52–66)	60–69	66 (63–73)
Eggs width	35 (29–38)	33	41 (37–42)	39 (33–47)	30–36	31 (27–36)

a Data taken from table in Bartoli et al. (2004).

b Calculated from data provided for lengths.

c No indication of which testes measured.

### Molecular remarks

**Figure 7.**
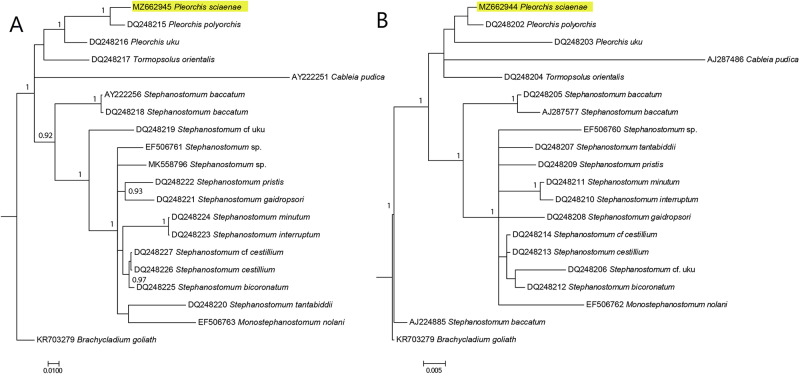
(A and B) Phylogenetic tree based on the Bayesian phylogenetic relationships of *Pleorchis sciaenae* specimen (this study, highlighted in yellow) and other species of the family Acanthocolpidae inferred from 28S (A) and 18S (B) rRNA sequences, available from GenBank (Supplementary Table S5). Clade posterior probability (>0.90) is indicated at nodes.

The results for the molecular sequences showed that our *P. sciaenae* specimen was distinct from both *P. polyorchis* and *P. uku* (Figures 7A and 7B), with 2.56% and 5.90% differences at 28S rRNA region and 0.4% and 2.3% differences at 18S rRNA region, respectively (Supplementary Tables S6 and Supplementary Tables S7). Although the level of difference between *P. sciaenae* and *P. polyorchis* is low for the 18S rRNA sequences, this level is similar for results obtained for various species of *Stephanostomum* (Supplementary Table S6). Phylogenetic analysis showed similar evolutionary relationship among the family of Acanthocolpidae at 28S and 18S rRNA regions (Figure 7A and 7B), although the 18S rRNA region generally has lower posterior probability. Our specimen was consistently grouped with other *Pleorchis* species at both 18S and 28S rRNA regions. These results support the morphological findings.

Molecular sequences did show that our specimens were different to the only two sequenced species: *P. polyorchis* from *Sciaena umbra* off the coast of Corsica, France, and *P. uku* from *Aprion virescens* (Lutjanidae) from Lizard Island, Australia (Bray et al., 2005). Both of these species are valid species and are quite distinct from each other morphologically (Bartoli et al., 2004). However, without more sequences to compare against, whether the sequences obtained in this study belong to an already described species, such as *P. sciaenae*, or a new species, cannot be determined. Thus, we have decided to err on the side of caution, with the morphological similarities and same host species, to identify the specimens collected in this study as *P. sciaenae* until further sequences are available.

## Discussion

This study has provided the first integrated description of the digenean parasites *O. diacanthi* and *P. sciaenae*, collected from *P. diacanthus* in Australian waters.

The family Cryptogonimidae has been reported from freshwater and marine teleosts, amphibians, and reptiles, with over 200 species, across 64 genera, recognized (Miller and Cribb, 2008b). Species belonging to the genus *Orientodiploproctodaeum* are distinguished from other genera of the Cryptogonomidae based on the presence of a large anterior collar surrounding the oral sucker, and the muscular lobe-like gonotyl residing immediately posterior to the ventral sucker (although large diversity of gonotyls is present in this family, most genera possess gonotyls that are immediately anterior to the ventral sucker) (Miller and Cribb, 2008b). *Orientodiploproctodaeum diacanthi* Bhutta and Khan 1970, type species of the genus, was first collected from *Pr. diacanthus* off the Karachi coast of Pakistan and described based on three specimens (Bray, 1987), from sciaenid, lutjanid and scombrid fish hosts from the Arabian Sea, the Bay of Bengal and the River Ganges. Taillebois et al. (2017) reported infections of an *Orientodiploproctodaeum* sp., from *P. diacanthus* from northern Australian waters, but did not officially identify the parasite, meaning that the current study represents the first hologenophore description, taxonomic identification and a new geographical location of *O. diacanthi* in Australian waters.

Apart from *O. diacanthi*, the only other species regarded as valid in the genus, *O. chinabutae* (Bray, 1987), was reported from the sciaenid *Nibea soldado* (Lacepède, 1802) in Thailand (Chinabut, 1984) and described from two specimens (Bray, 1987). Although *O. chinabutae* is notably larger and more elongate than *O. diacanthi*, it has been suggested that this could be a result of ontogenetic differences, rather than species-specific morphological characteristics (Bray, 1987). Large mature worms may in fact have no further need for sperm transfer and therefore resorbed the gonotyl and associated sac, suggesting that the distinguishing lack of a gonotyl and sac in *O. chinabutae* is not justified (Bray, 1987). However, the most recent morphological key to the genus lists species of *Orientodiploproctodaeum* as having either the presence or absence of a gonotyl (Miller and Cribb, 2008b). Further to this, Miller and Cribb (2008b) found no convincing morphological characters justifying sub-family divisions within Cryptogonimidae; however, they did stress the closeness of some groups of taxa based on their shared ecological and host preference (as seen in phylogenetic results in this study). It is therefore repeated here that until phylogenetic analyses are made more accessible and used more regularly to identify evolutionary lineages of genera, distinction based on morphological characters within and between cryptogonimid subfamilies is not viable (Miller and Cribb, 2008b). Genetic characterization of more representatives of *Orientodiploproctodaeum* from different host species and geographical locations is required to determine the validity of the current species identifications.

The family Acanthocolpidae infects marine teleosts and, occasionally, sea snakes, and represents a family of digeneans that is considered a ‘catch-all’ group, i.e. houses many genera that lack detailed descriptions and any molecular identification, with only a few species from 10 genera reporting integrated morphological and molecular description (Bray, 2005; Bray et al., 2005, 2007). *Pleorchis* is a well-defined genus within the Acanthocolpidae (Bray et al., 2005); however, identification of specimens to a species is more problematic. The species within *Pleorchis* are morphologically similar, with overlapping measurements, geographical ranges and host species, compounded by poor species descriptions based on few specimens. Most records of species of *Pleorchis* are from fish of the family Sciaenidae (Bray, 2005; Bray et al., 2005), however a number of species have been recently described from other fish families (Bartoli et al., 2004; Shaukat and Bilqees, 2006; Saxena et al., 2010; Bray and Justine, 2011; Gupta et al., 2011; Madhavi, 2011).

A total of 23 species from the genus *Pleorchis* have been described (Bartoli et al., 2004; Shaukat and Bilqees, 2006; Bilqees et al., 2010; Saxena et al., 2010; Gupta et al., 2011). Madhavi and Narasimhulu (1985) found significant intraspecific morphological variation in specimens of *P. sciaenae*, collected from four different host species in the Bay of Bengal, and suggested that this amount of variation within a single species could mean that characteristics traditionally used to differentiate closely related species may not be as reliable as they once were as they could be subject to intraspecific variation (keeping only the number of testes and the extent of vitellaria as distinguishing characters). Bray (1986) subsequently suggested that most species with about 44 testes should probably be considered synonyms of *P. sciaenae*. Some authors have followed this suggestion with some species (e.g., *P. ghanensis*; see Madhavi and Narasimhulu (1985)), but this has yet to be widely accepted (*P. ghanensis* was still considered a valid species by Bartoli et al. (2004)).

Bartoli et al. (2004) divided species into two groups, based on the number of testes: either approximately 44 or greater than 48, with the former group further subdivided based on the anterior extension of the vitelline fields. However, the fixation of specimens, especially in the early 1900s, often included flattening under pressure which can distort the internal structures (Cribb and Bray, 2010). Specimens of these ‘species’ need to be recollected and examined following a standard fixation technique to ensure that the distribution of the vitelline follicles can be used to differentiate the groups. Additionally, some descriptions and identifications of species were based on immature specimens (such as the *P. sciaenae* in Bray, 1986) which do not have fully formed vitelline fields, thus making determination difficult. Furthermore, many of the original descriptions of species were based on only a few specimens. Subsequent studies have shown that the collection of more specimens generally shows increased levels of intraspecific variation in many of the important taxonomic features (see Bartoli et al., 2004; Madhavi and Narasimhulu, 1985). Given the overlap in morphological features, the overlap in geographical distributions, the potential lack of specificity to host species, the lack of a systematic review of the genus, the apparent lack of agreement among authors and the lack of molecular sequences for members of the genus, the identification of most species remains in doubt.

The only identified *Pleorchis* species in Australian waters is *P. uku* from *Aprion virescens* (Lutjanidae), collected at Lizard Island, Great Barrier Reef (Bray et al., 2005). *Pleorchis uku*, however, is distinct from *P. sciaenae* in possessing 50–54 testes and appears to be specific to lutjanid fish from across a wide geographic distribution from Hawaii to China and New Caledonia and Lizard Island, with a potential report from a serranid fish from the Maldives (Bray and Justine, 2011). The results of the molecular analyses also supports *P. uku* as a distinct species from the specimens collected in this study, with *P. uku* the basal species to the clade of *P. polyorchis* and *P.sciaenae*.

*Pleorchis sciaenae* has been reported from *P. diacanthus* (as *P. ghanensis*; Bilqees, 1971; Bilqees, 1977; Madhavi and Narasumhulu, 1985) in Pakistan. Similar to *O. diacanthi*, Taillebois et al. (2017) reported infections of a *Pleorchis* sp. from *P. diacanthus* from northern Australian waters but did not officially identify the parasite. Therefore, the current study represents the first hologenophore description, taxonomic identification and a new geographical location of *P. sciaenae* in Australian waters.

Although digenean specimens collected from frozen hosts are not ideal for taxonomy (Cribb and Bray, 2010), the fish sampled in this study were collected for purposes other than parasite identification. In the reality of a world where access to fresh host specimens specifically for the collection of parasites to be processed for taxonomy (as per Cribb and Bray, 2010) becomes increasingly more difficult, we should consider the use of specimens from these ‘not ideal’ hosts as valuable in identifying future hosts of interest to be able to concentrate efforts. We acknowledge that the measurements of the specimens presented here need to be treated with an element of caution due to their collection method. However, with the incorporation of an integrated molecular approach, the results of this study will allow for future confirmation, or refutation, of the identifications provided.

The present study uses an integrated approach, combining morphological and molecular methods to identify two previously identified species *O. diacanthi* and *P. sciaenae* from *P. diacanthus* in Australian waters. The phylogenetic analyses presented in this study highlight the scarcity of genetic data available with many taxonomic descriptions of digeneans published prior to the development of molecular analysis techniques. Most species presented in the phylogenetic analyses lack sequences across a range of genes, leading to difficulties in deciphering the phylogenetic and evolutionary relationships of many species. Future research with more published genetic sequences will improve species-level identification of parasites in Australian waters, allowing for an improved understanding of parasitic influences on hosts and ecosystems.

## Supporting information

Porter et al. supplementary material 1 Porter et al. supplementary material

Porter et al. supplementary material 2 Porter et al. supplementary material

## Data Availability

All data produced for this study are provided in the manuscript.

## Supplementary material

The supplementary material for this article can be found at https://doi.org/10.1017/S0031182025000502.
