# Supplementary material for: Morphological and molecular characterization of the trematodes (Digenea: Acanthocolpidae and Cryptogonimidae) of the black-spotted croaker (Protonibea diacanthus) (Teleostei: Sciaenidae) in northern Australia
Source: Parasitology. 2025 Apr 4;152(4):453–68. doi: 10.1017/S0031182025000502 (PMC12186091; doi:10.1017/S0031182025000502)
Supplement: Porter et al. supplementary material 2 — Porter et al. supplementary material [file S0031182025000502sup002.docx]

Parasitology – Supplementary Material

**Morphological and molecular characterisation of the trematodes (Digenea: Acanthocolpidae and Cryptogonimidae) of the black-spotted croaker (*Protonibea diacanthus*) (Teleostei: Sciaenidae) in northern Australia**

Megan Porter* ^A, B^, Diane P. Barton ^A, B^, Xiaocheng Zhu ^C^ and Shokoofeh Shamsi ^A, B^

^A^ School of Agricultural, Environmental and Veterinary Sciences, Charles Sturt University, Wagga Wagga, NSW 2678, Australia.

^B^ Gulbali Institute, Charles Sturt University, Wagga Wagga, NSW 2678, Australia

^C^ NSW Department of Primary Industries and Regional Development (DPIRD), Wagga Wagga Agricultural Institute, Wagga Wagga, NSW 2650, Australia

* Corresponding author. Email: [mporter@csu.edu.au](mailto:mporter@csu.edu.au); ORCID: 0000-0003-1404-1391

**Supplementary Table S1.** Molecular sequences used in phylogenetic analyses for *Orientodiploproctodaeum diacanthi*. ID number refers to the genetic distance matrices in Supplementary Tables S2, S3 and S4.

| **ID** | **GenBank Accession** | **Parasite species** | **Gene** | **Host Species** | **Geographical Location** | **Reference** |
| --- | --- | --- | --- | --- | --- | --- |
| 1 | OQ888711 | *Orientodiploproctodaeum diacanthi* | 28S | *Protonibea diacanthus* | Northern Australia | This study |
| 2 | OQ888712 | *Orientodiploproctodaeum diacanthi* | 28S | *Protonibea diacanthus* | Northern Australia | This study |
| 3 | MN688143 | *Stephanostomum sp.* | 28S | *Freshwater fish* | Queensland | Miller and Adlard 2020 |
| 4 | MN688142 | *Stemmatostoma cribbi* | 28S | *Hephaestus fuliginosus* | Crystal Cascades: Queensland | Miller and Adlard 2020 |
| 5 | KF417632 | *Siphomutabilus raritas* | 28S | *Caesio cuning* | Lizard Island | Miller and Cribb 2013 |
| 6 | KF417631 | *Siphomutabilus gurukun* | 28S | *Caesio cuning* | Lizard Island | Miller and Cribb 2013 |
| 7 | EF116604 | *Retrovarium manteri* | 28S | *Lutjanus argentimaculatus* | Great Barrier Reef | Miller and Cribb 2007*a* |
| 8 | EF116605 | *Retrovarium brooksi* | 28S | *Lutjanus carponotatus* | Heron Island | Miller and Cribb 2007*a* |
| 9 | EF116606 | *Retrovarium gardneri* | 28S | *Lutjanus sebae* | Great Barrier Reef | Miller and Cribb 2007*a* |
| 10 | EF116607 | *Retrovarium mariae* | 28S | *Diagramma labiosum* | Heron Island | Miller and Cribb 2007*a* |
| 11 | EF116608 | *Retrovarium sablae* | 28S | *Aprion virescens* | Heron Island | Miller and Cribb 2007*a* |
| 12 | EF116610 | *Retrovarium synderi* | 28S | *Symphorus nematophorus* | Lizard Island | Miller and Cribb 2007*a* |
| 13 | EF116613 | *Retrovarium valdeparvum* | 28S | *Symphorus nematophorus* | Heron Island | Miller and Cribb 2007*a* |
| 14 | EF116612 | *Retrovarium exiguiformosum* | 28S | *Symphorus nematophorus* | Heron Island | Miller and Cribb 2007*a* |
| 15 | EF116611 | *Retrovarium formosum* | 28S | *Symphorus nematophorus* | Lizard Island | Miller and Cribb 2007*a* |
| 16 | EF116609 | *Retrovarium amplorificium* | 28S | *Symphorus nematophorus* | Heron Island | Miller and Cribb 2007*a* |
| 17 | EF116614 | *Retrovarium planum* | 28S | *Symphorus nematophorus* | Great Barrier Reef | Miller and Cribb 2007*a* |
| 18 | FJ788496 | *Adlardia novaecaledoniae* | 28S | *Nemipterus furcosus* | New Caledonia | Bray *et al*. 2009 |
| 19 | FJ554632 | *Adlardia novaecaledoniae* | 28S | *Nemipterus furcosus* | New Caledonia | Miller, Bray, Goiran, Justine and Cribb 2009 |
| 20 | AY222231 | *Caecincola parvulus* | 28S | *Micropterus salmoides* | Pascagoula River: Mississippi | Olsen *et al*. 2003 |
| 21 | EF566868 | *Beluesca longicolla* | 28S | *Plectorhinchus gibbosus* | Lizard Island | Miller and Cribb 2007*b* |
| 22 | HM187776 | *Varialvus jenae* | 28S | *Lutjanus carponotatus* | Lizard Island | Miller, Bray, Justine and Cribb, 2010 |
| 23 | HM187777 | *Varialvus lacertus* | 28S | *Lutjanus quinquelineatus* | Lizard Island | Miller, Bray, Justine and Cribb 2010 |
| 24 | HM187778 | *Varialvus charadrus* | 28S | *Lutjanus vitta* | Lizard Island | Miller, Bray, Justine and Cribb 2010 |
| 25 | EF428144 | *Caulanus thomasi* | 28S | *Lutjanus bohar* | Great Barrier Reef | Miller and Cribb 2007*a* |
| 26 | EF428146 | *Latuterus madivensis* | 28S | *Lutjanus bohar* | Rasdhoo: Maldives | Miller and Cribb 2007*a* |
| 27 | EF428145 | *Latuterus tkachi* | 28S | *Lutjanus bohar* | Lizard Island | Miller and Cribb 2007*a* |
| 28 | EU571262 | *Siphoderina virga* | 28S | *Lutjanus russelli* | North Stradbroke Island | Miller and Cribb 2008*a* |
| 29 | EF116615 | *Siphoderina territans* | 28S | *TBC* | TBC | Miller and Cribb 2007*a* |
| 30 | EU571265 | *Siphoderina quasispina* | 28S | *Lutjanus fulviflamma* | Heron Island | Miller and Cribb, 2008*a* |
| 31 | EU571263 | *Siphoderina jactus* | 28S | *Lutjanus fulviflamma* | Heron Island | Miller and Cribb 2007*a* |
| 32 | EU571264 | *Siphoderina infirma* | 28S | *Lutjanus russelli* | Lizard Island | Miller and Cribb 2008*a* |
| 33 | OM721660 | *Siphoderina hustoni* | 28S | *Lutjanus rivulatus* | Lizard Island | Martin and Cutmore 2022 |
| 34 | EU571261 | *Siphoderina grunnitus* | 28S | *Plectorhinchus gibbosus* | Lizard Island | Miller and Cribb 2008*a* |
| 35 | EU571266 | *Siphoderina subuterus* | 28S | *Lutjanus adetii* | Heron Island | Miller and Cribb 2008*a* |
| 36 | EU571267 | *Siphoderina poulini* | 28S | *Lutjanus argentimaculatus* | North Stradbroke Island | Miller and Cribb 2008*a* |
| 37 | KF417630 | *Metadena lutiani* | 28S | *Lutjanus bohar* | Great Barrier Reef | Miller and Cribb 2013 |
| 38 | FJ154902 | *Lobosorchis polygongylus* | 28S | *Lutjanus gobbus* | Lizard Island | Miller, Downie and Cribb, 2009 |
| 39 | FJ154901 | *Lobosorchis tibaldiae* | 28S | *Lutjanus carponotatus* | Heron Island | Miller, Downie and Cribb, 2009 |
| 40 | EF116616 | *Neometadena ovata* | 28S | *Lutjanus carponotatus* | Great Barrier Reef | Miller and Cribb 2007*a* |
| 41 | MH048926 | *Neometadena paucispina* | 28S | *Lutjanus fulviflamma* | North Stradbroke Island | Miller, Cutmore and Cribb 2018 |
| 42 | MK648274 | *Cryptogonimidae sp. 2* | 28S | *Centropomus undecimalis* | Salsipuedes: Tabasco | Pérez-Ponce de León and Hernández-Mena, 2019 |
| 43 | MK648275 | *Tabascotrema verai* | 28S | *Petenia splendida* | Metzabok: Chiapas | Pérez-Ponce de León and Hernández-Mena, 2019 |
| 44 | OL960047 | *Oligogonotylus manteri* | 28S | Cichilids | Nicaraguan Lakes | Santacruz *et al*. 2022 |
| 45 | MF491855 | *Timoniella imbutiforme* | 28S | *Dicentrarchus labrax* | Black Sea | Kvach *et al*. 2018 |
| 46 | MF983699 | *Timoniella imbutiforme* | 28S | *Dicentrarchus labrax* | Gulf of Gabès: Tunisia | Kacem, Blasco, Forondo and Miquel 2017 |
| 47 | MW000456^a^ | *Metagonimoides sp.* | 28S | Juga snails | Berry Creek: Oregon | Preston *et al*. 2020 |
| 48 | OQ888688 | *Orientodiploproctodaeum diacanthi* | ITS1 | *Protonibea diacanthus* | Northern Australia | This study |
| 49 | OQ888689 | *Orientodiploproctodaeum diacanthi* | ITS1 | *Protonibea diacanthus* | Northern Australia | This study |
| 50 | EU571255 | *Siphoderina hirastricta* | ITS1 | *Lutjanus argentimaculatus* | Lizard Island | Miller and Cribb 2008*a* |
| 51 | EU571254 | *Siphoderina poulini* | ITS1 | *Lutjanus argentimaculatus* | North Stradbroke Island | Miller and Cribb 2008*a* |
| 52 | EU571257 | *Siphoderina grunnitus* | ITS1 | *Plectorhinchus gibbosus* | Lizard Island | Miller and Cribb 2008*a* |
| 53 | EU571256 | *Siphoderina infirma* | ITS1 | *Lutjanus russelli* | Lizard Island | Miller and Cribb 2008*a* |
| 54 | EU571253 | *Siphoderina jactus* | ITS1 | *Lutjanus fulviflamma* | Heron Island | Miller and Cribb 2008*a* |
| 55 | EU571259 | *Siphoderina quasispina* | ITS1 | *Lutjanus fulviflamma* | Heron Island | Miller and Cribb 2008*a* |
| 56 | EF428143 | *Latuterus maldivensis* | ITS1 | *Lutjanus bohar* | Rasdhoo: Maldives | Miller and Cribb 2007*a* |
| 57 | EF428142 | *Latuterus tkachi* | ITS1 | *Lutjanus bohar* | Lizard Island | Miller and Cribb 2007*a* |
| 58 | EF428141 | *Caulanus thomasi* | ITS1 | *Lutjanus bohar* | Lizard Island | Miller and Cribb 2007*a* |
| 59 | EU571258 | *Siphoderina virga* | ITS1 | *Lutjanus russelli* | North Stradbroke Island | Miller and Cribb 2008*a* |
| 60 | EU571252 | *Siphoderina subuterus* | ITS1 | *Lutjanus adetii* | Heron Island | Miller and Cribb 2008*a* |
| 61 | HM187779 | *Varialvus jenae* | ITS1 | *Lutjanus carponotatus* | Lizard Island | Miller, Bray, Justine and Cribb 2010 |
| 62 | HM187780 | *Varialvus lacertus* | ITS1 | *Lutjanus quinquelineatus* | Lizard Island | Miller, Bray, Justine and Cribb 2010 |
| 63 | EF566871 | *Beluesca longicolla* | ITS1 | *Plectorhinchus gibbosus* | Heron Island | Miller and Cribb 2007*a* |
| 64 | EF566870 | *Beluesca littlewoodi* | ITS1 | *Plectorhinchus gibbosus* | Lizard Island | Miller and Cribb 2007*a* |
| 65 | EF116620 | *Siphoderina territans* | ITS1 | Unknown | Unknown | Miller and Cribb 2007*a* |
| 66 | KF417628 | *Siphomutabilus gurukun* | ITS1 | *Caesio cuning* | Lizard Island | Miller and Cribb 2013 |
| 67 | KF417629 | *Siphomutabilus raritas* | ITS1 | *Caesio cuning* | Lizard Island | Miller and Cribb 2013 |
| 68 | FJ154899 | *Lobosorchis tibaldiae* | ITS1 | *Lutjanus carponotatus* | Heron Island | Miller, Downie and Cribb 2009 |
| 69 | FJ154900 | *Lobosorchis polygongylus* | ITS1 | *Lutjanus gibbus* | Lizard Island | Miller, Downie and Cribb 2009 |
| 70 | FJ907332 | *Gynichthys diakidnus* | ITS1 | *Plectorhinchus gibbosus* | Lizard Island | Miller and Cribb 2009 |
| 71 | EF116626 | *Retrovarium exiguiformosum* | ITS1 | *Symphorus nematophorus* | Heron Island | Miller and Cribb 2007*a* |
| 72 | EF116625 | *Retrovarium formosum* | ITS1 | *Symphorus nematophorus* | Lizard Island | Miller and Cribb 2007*a* |
| 73 | EF116627 | *Retrovarium valdeparvum* | ITS1 | *Symphorus nematophorus* | Heron Island | Miller and Cribb 2007*a* |
| 74 | EF116623 | *Retrovarium amplorificium* | ITS1 | *Symphorus nematophorus* | Heron Island | Miller and Cribb 2007*a* |
| 75 | EF116624 | *Retrovarium synderi* | ITS1 | *Symphorus nematophorus* | Lizard Island | Miller and Cribb 2007*a* |
| 76 | EF116622 | *Retrovarium planum* | ITS1 | *Symphorus nematophorus* | Heron Island | Miller and Cribb 2007*a* |
| 77 | EF116618 | *Retrovarium marie* | ITS1 | *Diagramma labiosum* | Heron Island | Miller and Cribb 2007*a* |
| 78 | HM187781 | *Varialvus charadrus* | ITS1 | *Lutjanus vitta* | Lizard Island | Miller, Bray, Justine and Cribb 2010 |
| 79 | EF566869 | *Chelediadema marjoriae* | ITS1 | *Diagramma labiosum* | Heron Island | Miller and Cribb 2007*a* |
| 80 | JX023371 | *Tabascotrema verai* | ITS1 | *Petenia splendida* | Lake Petén Itzá: Guatemala | Razo-Mendivil *et al*. 2015 |
| 81 | JX023354 | *Tabascotrema verai* | ITS1 | *Petenia splendida* | Pantanos de Centla wetlands: Tabasco | Razo-Mendivil *et al*. 2015 |
| 82 | JX023362 | *Tabascotrema verai* | ITS1 | *Petenia splendida* | Chilapa: Tabasco | Razo-Mendivil *et al*. 2015 |
| 83 | JX023344 | *Tabascotrema verai* | ITS1 | *Petenia splendida* | Candelaria River: Mexico | Razo-Mendivil *et al*. 2015 |
| 84 | HM056034 | *Cryptogonimidae sp. 1* | ITS1 | *Lutjanus kasmira* | Rasdhoo: Maldives | Miller *et al*. 2010 |
| 85 | HM056033 | *Euryakaina manilensis* | ITS1 | *Lutjanus vitta* | Luzon Island: Phillipines | Miller, Adlard, Bray, Justine and Cribb 2010 |
| 86 | HM056032 | *Euryakaina marina* | ITS1 | *Lutjanus carponotatus* | Lizard Island | Miller, Adlard, Bray, Justine and Cribb 2010 |
| 87 | EU662192 | *Oligogonotylus manteri* | ITS1 | *Cichlasoma urophthalmus* | Papalopapan River:  Veracruz | Razo-Mendivil *et al*. 2008 |
| 88 | EU662195 | *Oligogonotylus manteri* | ITS1 | *Cichlasoma urophthalmus* | Crooked Tree Lagoon: Belize | Razo-Mendivil *et al*. 2008 |
| 89 | EU662191 | *Oligogonotylus manteri* | ITS1 | *Cichlasoma urophthalmus* | River Papalopapan: Vercruz | Razo-Mendivil et al. 2008 |
| 90 | EU662197 | *Oligogonotylus mayae* | ITS1 | *Cichlasoma urophthalmus* | Ría Celestun Biosphere Reserve: Mexico | Razo-Mendivil *et al*. 2008 |
| 91 | MF491842 | *Timoniella imbutiformis* | ITS1 | *Pomatoschistus microps* | St Nazaire Lagoon: Mediterranean Sea | Kvach *et al*. 2018 |
| 92 | MF491832 | *Timoniella imbutiformis* | ITS1 | *Pomatoschistus microps* | Salzhaff: Baltic Sea | Kvach *et al*. 2018 |
| 93 | MG383513 | *Acanthostomum americanum* | ITS1 | *Cichlasoma urophthalmus* | Ria Celestun Biosphere Reserve: Mexico | Martínez-Aquino *et al*. 2017 |
| 94 | AY245702^b^ | *Dexiogonimus ciureanus* | ITS1 | *Phalacrocorax carbo* | Israel | Dzikowski *et al*. 2004 |
| 95 | OQ888737 | *Orientodiploproctodaeum diacanthi* | ITS2 | *Protonibea diacanthus* | Northern Australia | This study |
| 96 | OQ888738 | *Orientodiploproctodaeum diacanthi* | ITS2 | *Protonibea diacanthus* | Northern Australia | This study |
| 97 | OQ888739 | *Orientodiploproctodaeum diacanthi* | ITS2 | *Protonibea diacanthus* | Northern Australia | This study |
| 98 | MN688140 | *Stematostoma cribbi* | ITS2 | *Hephaestus fuliginosus* | Crystal Cascades: Queensland | Miller and Adlard 2020 |
| 99 | MN688141 | *Stematostomma pearsoni* | ITS2 | *Posticobia brazieri* | Brisbane | Miller and Adlard 2020 |
| 100 | EU571259 | *Siphoderina quasispina* | ITS2 | *Lutjanus fulviflamma* | Great Barrier Reef | Miller and Cribb 2008*a* |
| 101 | EU571257 | *Siphoderina grunnitus* | ITS2 | *Plectorhinchus* | Lizard Island | Miller and Cribb 2008*a* |
| 102 | FJ154899 | *Lobosorchis tibaldiae* | ITS2 | *Neoglyphidodon melas* | Heron Island | Miller, Downie and Cribb 2009 |
| 103 | MH025623 | *Cryptocotyle lata* | ITS2 | *Anas platyrhynchos* | Bolshaya Ussurka River: Russia | Tatonova and Besprozvannykh 2019 |
| 104 | MH025622 | *Cryptocotyle lata* | ITS2 | *Anas platyrhynchos* | Bolshaya Ussurka River: Russia | Tatonova and Besprozvannykh 2019 |
| 105 | MK877249 | *Erschoviorchis anuiensis* | ITS2 | *Cairina moschata* | Anyui River: Khabarovsk Region | Tatonova *et al*. 2020 |
| 106 | MK877248 | *Erschoviorchis anuiensis* | ITS2 | *Cairina moschata* | Anyui River: Khabarovsk Region | Tatonova *et al*. 2020 |
| 107 | MK877247 | *Erschoviorchis anuiensis* | ITS2 | *Cairina moschata* | Anyui River: Khabarovsk Region | Tatonova *et al*. 2020 |
| 108 | MK877246 | *Erschoviorchis anuiensis* | ITS2 | *Cairina moschata* | Anyui River: Khabarovsk Region | Tatonova *et al*. 2020 |
| 109 | MK877245 | *Erschoviorchis anuiensis* | ITS2 | *Cairina moschata* | Anyui River: Khabarovsk Region | Tatonova *et al*. 2020 |
| 110 | MW544139 | *Cryptocotyle lingua* | ITS2 | Gadidae | English Channel: France | Duflot *et al*. 2021 |
| 111 | MW544131 | *Cryptocotyle lingua* | ITS2 | Gadidae | English Channel: France | Duflot *et al*. 2021 |
| 112 | MW544125 | *Cryptocotyle lingua* | ITS2 | Gadidae | English Channel: France | Duflot *et al*. 2021 |
| 113 | MW544115 | *Cryptocotyle lingua* | ITS2 | Gadidae | English Channel: France | Duflot *et al*. 2021 |
| 114 | MW544134 | *Cryptocotyle lingua* | ITS2 | Gadidae | English Channel: France | Duflot *et al*. 2021 |
| 115 | MW544138 | *Cryptocotyle lingua* | ITS2 | Gadidae | English Channel: France | Duflot *et al*. 2021 |
| 116 | MW544124 | *Cryptocotyle lingua* | ITS2 | Gadidae | English Channel: France | Duflot *et al*. 2021 |
| 117 | MW544136 | *Cryptocotyle lingua* | ITS2 | Gadidae | English Channel: France | Duflot *et al*. 2021 |
| 118 | MW544135 | *Cryptocotyle lingua* | ITS2 | Gadidae | English Channel: France | Duflot *et al*. 2021 |
| 119 | MW544133 | *Cryptocotyle lingua* | ITS2 | Gadidae | English Channel: France | Duflot *et al*. 2021 |
| 120 | MW544120 | *Cryptocotyle lingua* | ITS2 | Gadidae | English Channel: France | Duflot *et al*. 2021 |
| 121 | MW544132 | *Cryptocotyle lingua* | ITS2 | Gadidae | English Channel: France | Duflot *et al*. 2021 |
| 122 | MW544129 | *Cryptocotyle lingua* | ITS2 | Gadidae | English Channel: France | Duflot *et al*. 2021 |
| 123 | MW544112 | *Cryptocotyle lingua* | ITS2 | Gadidae | English Channel: France | Duflot *et al*. 2021 |
| 124 | MT268114 | *Parapleurolophocercous* sp. | ITS2 | Unidentified snails | Yen Bai Province | Nguyen *et al*. 2021 |
| 125 | MF438073 | *Apophallus* sp. | ITS2 | *Chondrostoma nasus* | River Danube: Hungary | Sandor *et al*. 2017 |
| 126 | MF438072 | *Apophallus* sp. | ITS2 | *Chondrostoma nasus* | River Danube: Hungary | Sandor *et al*. 2017 |
| 127 | MF438051 | *Apophallus* sp. | ITS2 | *Squalius cephalus* | River Danube: Hungary | Sandor *et al*. 2017 |
| 128 | MF438050 | *Apophallus* sp. | ITS2 | *Squalius cephalus* | River Danube: Hungary | Sandor *et al*. 2017 |
| 129 | MF438075 | *Apophallus* sp. | ITS2 | *Scardinius erythrophthalmus* | Lake Balaton: Siófok | Sandor *et al*. 2017 |
| 130 | MF438069 | *Apophallus muehlingi* | ITS2 | *Abramis brama* | Lake Balaton: Siófok | Sandor *et al*. 2017 |
| 131 | MF438066 | *Apophallus muehlingi* | ITS2 | *Blicca bjoerkna* | River Danube: Keszthely | Sandor *et al*. 2017 |
| 132 | MF438065 | *Apophallus muehlingi* | ITS2 | *Abramis brama* | Lake Balaton: Keszthely | Sandor *et al*. 2017 |
| 133 | MF438053 | *Apophallus muehlingi* | ITS2 | Commercial chickens | Hungary | Sandor *et al*. 2017 |
| 134 | MF438064 | *Apophallus muehlingi* | ITS2 | *Abramis brama* | Lake Balaton: Keszthely | Sandor *et al*. 2017 |
| 135 | MF438052 | *Apophallus muehlingi* | ITS2 | Commercial chickens | Hungary | Sandor *et al*. 2017 |
| 136 | MF438049 | *Apophallus muehlingi* | ITS2 | *Lithoglyphus naticoides* | Lake Balaton: Tihany | Sandor *et al*. 2017 |
| 137 | MF438054 | *Apophallus muehlingi* | ITS2 | Commercial chickens | Hungary | Sandor *et al*. 2017 |
| 138 | MF438062 | *Apophallus muehlingi* | ITS2 | *Lithoglyphus naticoides* | Lake Balaton: Keszthely | Sandor *et al*. 2017 |
| 139 | MF438068 | *Apophallus muehlingi* | ITS2 | *Abramis brama* | Lake Balaton: Siófok | Sandor *et al*. 2017 |
| 140 | MF407173 | *Metagonimus pusillus* | ITS2 | *Anas platyrhynchos* | Russia | Tatanova *et al*. 2018 |
| 141 | MF407172 | *Metagonimus pusillus* | ITS2 | *Anas platyrhynchos* | Russia | Tatanova *et al*. 2018 |
| 142 | MZ825158^c^ | *Heterophyidae* sp. | ITS2 | *Anentome helena* | Mae Rim District: Chiang Mai Province | Chomchoei *et al*. 2022 |

^a^ Outgroup for 28S; ^b^ Outgroup for ITS1; ^c^ Outgroup for ITS2

**Supplementary Table S2**. Estimates of evolutionary divergence between sequences of the 28S rRNA region of our *Orientodiploproctodaeum diacanthi* sample compared to close related species from the family of Cryptogonimidae in GenBank. Please refer to Table S1 for ID number.

**Supplementary Table S3**. Estimates of evolutionary divergence between sequences of the ITS1 region of our *Orientodiploproctodaeum diacanthi* sample compared to close related species from the family of Cryptogonimidae in GenBank. Please refer to Table S1 for ID number

**Supplementary Table S4**. Estimates of evolutionary divergence between sequences of the ITS2 region of our *Orientodiploproctodaeum diacanthi* sample compared to close related species from the family of Cryptogonimidae in GenBank. Please refer to Table S1 for ID number.

Data available here:

**Supplementary Table S5**. Molecular sequences used in phylogenetic analyses of *Pleorchis sciaenae*. ID number refers to the genetic distance matrices in Supplementary Tables S6 and S7.

| **ID** | **GenBank Accession** | **Parasite species** | **Gene** | **Host Species** | **Geographical Location** | **Reference** |
| --- | --- | --- | --- | --- | --- | --- |
| 1 | JF3225_1 | *Pleorchis sciaenae* | 18S | *Protonibea diacanthus* | Northern Australia | This study |
| 2 | DQ248202 | *Pleorchis polyorchis* | 18S | *Sciaena umbra* | Corsica, France | Bray *et al*. 2005 |
| 3 | DQ248203 | *Pleorchis uku* | 18S | *Aprion virescens* | Lizard Is., Australia | Bray *et al*. 2005 |
| 4 | DQ248204 | *Tormopsolus orientalis* | 18S | *Seriola dumerli* | Corsica, France | Bray *et al*. 2005 |
| 5 | EF506762 | *Monostephanostomum nolani* | 18S | *Carangoides plagiotaenia* | Australia | Bray *et al*. 2007 |
| 6 | AJ224885 | *Stephanostomum baccatum* | 18S | *Eutrigla gurnardus* | Not stated | Fernandez *et al*. 1998 |
| 7 | AJ287577 | *Stephanostomum baccatum* | 18S | *Eutrigla gurnardus* | Not stated | Littlewood and Olsen 2001 |
| 8 | DQ248205 | *Stephanostomum baccatum* | 18S | *Hippoglossoides hippoglossus* | North Sea, United Kingdom | Bray *et al*. 2005 |
| 9 | DQ248212 | *Stephanostomum bicoronatum* | 18S | *Sciaena umbra* | Corsica, France | Bray *et al*. 2005 |
| 10 | DQ248213 | *Stephanostomum cestillium* | 18S | *Lophius piscatorius* | Corsica, France | Bray *et al*. 2005 |
| 11 | DQ248208 | *Stephanostomum gaidropsori* | 18S | *Gaidropsarus mediterraneus* | Marseille, France | Bray *et al*. 2005 |
| 12 | DQ248210 | *Stephanostomum interruptum* | 18S | *Menticirrhus americanus* | Gulf of Mexico, USA | Bray *et al*. 2005 |
| 13 | DQ248211 | *Stephanostomum minutum* | 18S | *Uranoscopus scaber* | Corsica, France | Bray *et al*. 2005 |
| 14 | DQ248209 | *Stephanostomum pristis* | 18S | *Phycis phycis* | Corsica, France | Bray *et al*. 2005 |
| 15 | DQ248207 | *Stephanostomum tantabiddii* | 18S | *Carangoides fulviguttatus* | Ningaloo Reef, Australia | Bray *et al*. 2005 |
| 16 | DQ248214 | *Stephanostomum cf cestillium* | 18S | *Zeus faber* | Corsica, France | Bray *et al*. 2005 |
| 17 | DQ248206 | *Stephanostomum cf. uku* | 18S | *Aprion virescens* | Lizard Is., Australia | Bray *et al*. 2005 |
| 18 | EF506760 | *Stephanostomum* sp. | 18S | *Plectropomus leopardus* | Australia | Bray *et al*. 2007 |
| 19 | AJ287486 | *Cableia pudica* | 18S | *Cantherhines pardalis* | Not stated | Littlewood and Olsen 2001 |
| 20 | KR703279 | *Brachycladium goliath* | 18S | *Balaneoptera acutorostrata* | United Kingdom | Briscoe *et al*. 2016 |
| 21 | JF3225_1 | *Pleorchis sciaenae* | 28S | *Protonibea diacanthus* | Northern Australia | This study |
| 22 | DQ248215 | *Pleorchis polyorchis* | 28S | *Sciaena umbra* | Corsica, France | Bray *et al*. 2005 |
| 23 | DQ248216 | *Pleorchis uku* | 28S | *Aprion virescens* | Lizard Is., Australia | Bray *et al*. 2005 |
| 24 | DQ248217 | *Tormopsolus orientalis* | 28S | *Seriola dumerli* | Corsica, France | Bray *et al*. 2005 |
| 25 | EF506763 | *Monostephanostomum nolani* | 28S | *Carangoides plagiotaenia* | Australia | Bray *et al*. 2007 |
| 26 | DQ248218 | *Stephanostomum baccatum* | 28S | *Hippoglossoides hippoglossus* | North Sea, United Kingdom | Bray *et al*. 2005 |
| 27 | AY222256 | *Stephanostomum baccatum* | 28S | *Eutrigla gurnardus* | United Kingdom | Olsen *et al*. 2003 |
| 28 | DQ248225 | *Stephanostomum bicoronatum* | 28S | *Sciaena umbra* | Corsica, France | Bray *et al*. 2005 |
| 29 | DQ248226 | *Stephanostomum cestillium* | 28S | *Lophius piscatorius* | Corsica, France | Bray *et al*. 2005 |
| 30 | DQ248221 | *Stephanostomum gaidropsori* | 28S | *Gaidropsarus mediterraneus* | Marseille, France | Bray *et al*. 2005 |
| 31 | DQ248223 | *Stephanostomum interruptum* | 28S | *Menticirrhus americanus* | Gulf of Mexico, USA | Bray *et al*. 2005 |
| 32 | DQ248224 | *Stephanostomum minutum* | 28S | *Uranoscopus scaber* | Corsica, France | Bray *et al*. 2005 |
| 33 | DQ248222 | *Stephanostomum pristis* | 28S | *Phycis phycis* | Corsica, France | Bray *et al*. 2005 |
| 34 | DQ248220 | *Stephanostomum tantabiddii* | 28S | *Carangoides fulviguttatus* | Ningaloo Reef, Australia | Bray *et al*. 2005 |
| 35 | DQ248227 | *Stephanostomum cf cestillium* | 28S | *Zeus faber* | Corsica, France | Bray *et al*. 2005 |
| 36 | DQ248219 | *Stephanostomum cf uku* | 28S | *Aprion virescens* | Lizard Is., Australia | Bray *et al*. 2005 |
| 37 | MK558796 | *Stephanostomum* sp*.* | 28S | *Syacium papillosum* | Mexico | Vidal-Martinez *et al*. 2019 |
| 38 | EF506761 | *Stephanostomum* sp. | 28S | *Plectropomus leopardus* | Australia | Bray *et al*. 2007 |
| 39 | AY222251 | *Cableia pudica* | 28S | *Cantherhines pardalis* | Australia | Olsen *et al*. 2003 |
| 40 | KR703279 | *Brachycladium goliath* | 28S | *Balaneoptera acutorostrata* | United Kingdom | Briscoe *et al*. 2016 |

**Supplementary Table S6**. Estimates of evolutionary divergence between sequences of the 18S rRNA region of our *Pleorchis sciaenae* sample compared to close related species from the family of Acanthocolpidae in GenBank, shown as p-difference (below the diagonal) and number of differences (above the diagonal). Please refer to Table S5 for ID number.

| **ID** | **1** | **2** | **3** | **4** | **5** | **6** | **7** | **8** | **9** | **10** | **11** | **12** | **13** | **14** | **15** | **16** | **17** | **18** | **19** |
| --- | --- | --- | --- | --- | --- | --- | --- | --- | --- | --- | --- | --- | --- | --- | --- | --- | --- | --- | --- |
| **1** |  |  |  |  |  |  |  |  |  |  |  |  |  |  |  |  |  |  |  |
| **2** | 0.004 |  |  |  |  |  |  |  |  |  |  |  |  |  |  |  |  |  |  |
| **3** | 0.023 | 0.027 |  |  |  |  |  |  |  |  |  |  |  |  |  |  |  |  |  |
| **4** | 0.017 | 0.016 | 0.033 |  |  |  |  |  |  |  |  |  |  |  |  |  |  |  |  |
| **5** | 0.034 | 0.033 | 0.053 | 0.038 |  |  |  |  |  |  |  |  |  |  |  |  |  |  |  |
| **6** | 0.016 | 0.017 | 0.032 | 0.020 | 0.042 |  |  |  |  |  |  |  |  |  |  |  |  |  |  |
| **7** | 0.030 | 0.033 | 0.046 | 0.037 | 0.047 | 0.034 |  |  |  |  |  |  |  |  |  |  |  |  |  |
| **8** | 0.025 | 0.028 | 0.041 | 0.032 | 0.042 | 0.030 | 0.005 |  |  |  |  |  |  |  |  |  |  |  |  |
| **9** | 0.023 | 0.026 | 0.044 | 0.027 | 0.023 | 0.030 | 0.031 | 0.026 |  |  |  |  |  |  |  |  |  |  |  |
| **10** | 0.021 | 0.023 | 0.042 | 0.027 | 0.021 | 0.027 | 0.028 | 0.023 | 0.002 |  |  |  |  |  |  |  |  |  |  |
| **11** | 0.026 | 0.028 | 0.047 | 0.030 | 0.031 | 0.032 | 0.033 | 0.028 | 0.015 | 0.012 |  |  |  |  |  |  |  |  |  |
| **12** | 0.030 | 0.033 | 0.048 | 0.037 | 0.030 | 0.037 | 0.037 | 0.032 | 0.016 | 0.014 | 0.023 |  |  |  |  |  |  |  |  |
| **13** | 0.027 | 0.031 | 0.046 | 0.034 | 0.027 | 0.034 | 0.034 | 0.030 | 0.014 | 0.011 | 0.021 | 0.002 |  |  |  |  |  |  |  |
| **14** | 0.025 | 0.027 | 0.046 | 0.031 | 0.027 | 0.031 | 0.032 | 0.027 | 0.014 | 0.011 | 0.018 | 0.022 | 0.020 |  |  |  |  |  |  |
| **15** | 0.025 | 0.027 | 0.042 | 0.028 | 0.027 | 0.028 | 0.026 | 0.021 | 0.011 | 0.009 | 0.016 | 0.020 | 0.017 | 0.015 |  |  |  |  |  |
| **16** | 0.021 | 0.023 | 0.042 | 0.027 | 0.021 | 0.027 | 0.028 | 0.023 | 0.002 | 0.000 | 0.012 | 0.014 | 0.011 | 0.011 | 0.009 |  |  |  |  |
| **17** | 0.027 | 0.027 | 0.046 | 0.026 | 0.030 | 0.032 | 0.030 | 0.025 | 0.011 | 0.011 | 0.021 | 0.025 | 0.022 | 0.020 | 0.018 | 0.011 |  |  |  |
| **18** | 0.037 | 0.038 | 0.055 | 0.039 | 0.037 | 0.038 | 0.039 | 0.034 | 0.023 | 0.021 | 0.028 | 0.032 | 0.030 | 0.023 | 0.025 | 0.021 | 0.023 |  |  |
| **19** | 0.054 | 0.054 | 0.073 | 0.055 | 0.071 | 0.062 | 0.069 | 0.064 | 0.064 | 0.064 | 0.069 | 0.070 | 0.068 | 0.069 | 0.070 | 0.064 | 0.065 | 0.073 |  |

**Supplementary Table S7**. Estimates of evolutionary divergence between sequences of the 28S rRNA region of our *Pleorchis sciaenae* sample compared to close related species from the family of Acanthocolpidae in GenBank, shown as p-difference (below the diagonal) and number of differences (above the diagonal). Please refer to Table S5 for ID number.

| **ID** | **21** | **22** | **23** | **24** | **25** | **26** | **27** | **28** | **29** | **30** | **31** | **32** | **33** | **34** | **35** | **36** | **37** | **38** | **39** |
| --- | --- | --- | --- | --- | --- | --- | --- | --- | --- | --- | --- | --- | --- | --- | --- | --- | --- | --- | --- |
| **21** |  |  |  |  |  |  |  |  |  |  |  |  |  |  |  |  |  |  |  |
| **22** | 0.026 |  |  |  |  |  |  |  |  |  |  |  |  |  |  |  |  |  |  |
| **23** | 0.059 | 0.055 |  |  |  |  |  |  |  |  |  |  |  |  |  |  |  |  |  |
| **24** | 0.079 | 0.073 | 0.065 |  |  |  |  |  |  |  |  |  |  |  |  |  |  |  |  |
| **25** | 0.122 | 0.121 | 0.114 | 0.106 |  |  |  |  |  |  |  |  |  |  |  |  |  |  |  |
| **26** | 0.095 | 0.088 | 0.073 | 0.080 | 0.092 |  |  |  |  |  |  |  |  |  |  |  |  |  |  |
| **27** | 0.095 | 0.088 | 0.073 | 0.080 | 0.092 | 0.000 |  |  |  |  |  |  |  |  |  |  |  |  |  |
| **28** | 0.097 | 0.104 | 0.086 | 0.086 | 0.059 | 0.076 | 0.076 |  |  |  |  |  |  |  |  |  |  |  |  |
| **29** | 0.096 | 0.102 | 0.085 | 0.085 | 0.058 | 0.072 | 0.072 | 0.003 |  |  |  |  |  |  |  |  |  |  |  |
| **30** | 0.108 | 0.107 | 0.092 | 0.094 | 0.063 | 0.076 | 0.076 | 0.036 | 0.032 |  |  |  |  |  |  |  |  |  |  |
| **31** | 0.121 | 0.125 | 0.108 | 0.108 | 0.087 | 0.094 | 0.094 | 0.038 | 0.037 | 0.057 |  |  |  |  |  |  |  |  |  |
| **32** | 0.121 | 0.125 | 0.108 | 0.108 | 0.087 | 0.094 | 0.094 | 0.038 | 0.037 | 0.057 | 0.000 |  |  |  |  |  |  |  |  |
| **33** | 0.105 | 0.098 | 0.090 | 0.090 | 0.060 | 0.073 | 0.073 | 0.033 | 0.030 | 0.038 | 0.055 | 0.055 |  |  |  |  |  |  |  |
| **34** | 0.121 | 0.118 | 0.101 | 0.101 | 0.076 | 0.094 | 0.094 | 0.049 | 0.050 | 0.057 | 0.073 | 0.073 | 0.062 |  |  |  |  |  |  |
| **35** | 0.096 | 0.102 | 0.085 | 0.085 | 0.058 | 0.072 | 0.072 | 0.003 | 0.000 | 0.032 | 0.037 | 0.037 | 0.030 | 0.050 |  |  |  |  |  |
| **36** | 0.107 | 0.107 | 0.091 | 0.099 | 0.086 | 0.078 | 0.078 | 0.059 | 0.060 | 0.072 | 0.078 | 0.078 | 0.069 | 0.075 | 0.060 |  |  |  |  |
| **37** | 0.102 | 0.105 | 0.086 | 0.086 | 0.069 | 0.072 | 0.072 | 0.033 | 0.030 | 0.040 | 0.055 | 0.055 | 0.042 | 0.062 | 0.030 | 0.059 |  |  |  |
| **38** | 0.105 | 0.109 | 0.089 | 0.094 | 0.071 | 0.089 | 0.089 | 0.031 | 0.030 | 0.046 | 0.055 | 0.055 | 0.047 | 0.053 | 0.030 | 0.069 | 0.040 |  |  |
| **39** | 0.165 | 0.161 | 0.155 | 0.158 | 0.170 | 0.155 | 0.155 | 0.164 | 0.160 | 0.164 | 0.169 | 0.169 | 0.163 | 0.169 | 0.160 | 0.156 | 0.155 | 0.161 |  |
